# Paternal care effects outweigh gamete-mediated and personal environment effects during the transgenerational estimation of risk in fathead minnows

**DOI:** 10.1186/s12862-021-01919-1

**Published:** 2021-10-11

**Authors:** Denis Meuthen, Maud C. O. Ferrari, Douglas P. Chivers

**Affiliations:** 1 Department of Biology, University of Saskatchewan, 112 Science Place, Saskatoon, SK S7N 5E2 Canada; 2 Evolutionary Biology, Bielefeld University, Konsequenz 45, 33615 Bielefeld, Germany; 3 Department of Veterinary Biomedical Sciences, WCVM, University of Saskatchewan, 52 Campus Drive, Saskatoon, SK S7N 5B4 Canada

**Keywords:** *Pimephales promelas*, Alarm cues, Transgenerational plasticity, Phenotypic plasticity, Risk assessment, Risk allocation, Non-genetic inheritance, Parental care, Maternal effects, Paternal effects

## Abstract

**Background:**

Individuals can estimate risk by integrating prenatal with postnatal and personal information, but the relative importance of different information sources during the transgenerational response is unclear. The estimated level of risk can be tested using the cognitive rule of risk allocation, which postulates that under consistent high-risk, antipredator efforts should decrease so that individual metabolic requirements can be satisfied. Here we conduct a comprehensive study on transgenerational risk transmission by testing whether risk allocation occurs across 12 treatments that consist of different maternal, paternal, parental care (including cross-fostering) and offspring risk environment combinations in the fathead minnow *Pimephales promelas*, a small cyprinid fish with alloparental care. In each risk environment, we manipulated perceived risk by continuously exposing individuals from birth onwards to conspecific alarm cues or a control water treatment. Using 2810 1-month old individuals, we then estimated shoaling behaviour prior to and subsequent to a novel mechanical predator disturbance.

**Results:**

Overall, shoals estimating risk to be high were denser during the prestimulus period, and, following the risk allocation hypothesis, resumed normal shoaling densities faster following the disturbance. Treatments involving parental care consistently induced densest shoals and greatest levels of risk allocation. Although prenatal risk environments did not relate to paternal care intensity, greater care intensity induced more risk allocation when parents provided care for their own offspring as opposed to those that cross-fostered fry. In the absence of care, parental effects on shoaling density were relatively weak and personal environments modulated risk allocation only when parental risk was low.

**Conclusions:**

Our study highlights the high relative importance of parental care as opposed to other information sources, and its function as a mechanism underlying transgenerational risk transmission.

**Supplementary Information:**

The online version contains supplementary material available at 10.1186/s12862-021-01919-1.

## Background

In a heterogeneous, ever-changing world where risk fluctuates temporally and spatially [1], individuals have to carefully balance antipredator strategies that are costly [2, 3] but increase survival [4, 5], with other fitness-enhancing activities such as foraging and mating [6]. Hence, to maximize individual fitness, individuals need to accurately estimate the level of risk to which they are exposed. For this purpose, they derive information from various risk cues that are present in their environment [7]. Additionally, they can obtain risk-related information from previous generations and integrate it through transgenerational effects. Transgenerational plasticity, which evolves only when environmental change is slower than generation time [8], or when future environmental change can be accurately predicted [9], is widespread [10]. Due to its relevance in allowing organisms to cope with climate change [11, 12], and in reducing populations’ extinction risk in a changing world [13], researchers have been pushing for a better understanding of this phenomenon [10, 12].

However, remarkably little is known about the relative importance of risk information from different sources during the transgenerational response. First, risk information can be transmitted to the next generation through prefertilization parental effects, i.e., risk-induced changes in maternal [14–19] or paternal gametes [16, 20] but the relative importance of maternal and paternal risk information remains an open question [16, 21]. Second, risk-related cues can be communicated to offspring through postfertilization parental effects such as altered parental care [22–25]. As prenatal and postnatal effects can be co-adapted [26], revealing their individual and interactive effects is important for understanding evolutionary processes [27–29]. Third, individuals can obtain personal risk information themselves, which can interact with parental information in different ways, dependent on the level of environmental autocorrelation, cue reliability and the accuracy of transgenerational inheritance [30–32]. Taken together, there is a clear need for more comprehensive studies that reveal the relative importance of risk-related prefertilization parental effects, postfertilization parental effects and personal risk information [21, 33, 34].

Here, we study the transmission and integration of risk from different sources across generations in the fathead minnow *Pimephales promelas* [35]. This cyprinid is a common prey fish widespread across North American rivers and lakes [36]. After shoaling as juveniles, adult males become territorial and provide intensive alloparental care to clutches [37]. Males also provide alloparental care to adopted eggs as doing so increases their attractiveness to females [38] but they do discriminate between own and adopted clutches, providing less care to the latter [39]. In this species, parental care consists of clutch defense from predators, and egg cleaning by constant rubbing and mouthing [36, 39]. *P. promelas* experiences heterogeneous predation risk across populations [40, 41] but within populations, the level of predation risk is often stable. This is because first, like other cyprinids, *P. promelas* is a fish without constitutive morphological defenses such as spiny fish rays or sturdy scales, making it vulnerable to a wide variety of general predators throughout ontogeny [42]. Second, they are a non-migratory species that often inhabit small lakes and ponds where predator presence is consistent over time [36]. Third, lifespans of predators often cover multiple *P. promelas* generations. For example, a common sympatric predator of minnows is the northern pike *Esox lucius* [43], whose maximum age ranges between 8 and 15 years [44]. This means that *P. promelas* with their 6-month generation time can be exposed to the same predator individuals for 16–30 generations, which suggests that predator presence can be reliably predicted across multiple generations. *P. promelas* is also a well-established model system for studying risk assessment [45, 46] and antipredator phenotypic plasticity [47–49], including transgenerational responses [50].

**Fig. 1:**
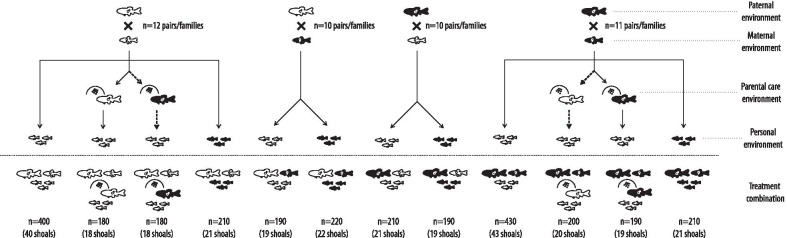
Breeding design aiming to capture independent and cumulative impacts of paternal, maternal, parental care and personal environments on risk assessment in the offspring generation. Black fish indicate individuals that were from birth onwards exposed to conspecific alarm cues, white fish refer to the ones that instead received a water control treatment. Dashed lines indicate the two treatments that resulted from cross-fostering. The bottom row below the dotted line introduces the cumulative figures that represent the specific treatment combination. Sample sizes of families, individuals and shoals that were analyzed are stated throughout

Using continuous exposure from birth onwards to either conspecific alarm cues (high-risk) or a water control (low-risk), in a split-clutch design, we crossed risk levels across maternal, paternal, parental care (including cross-fostering) and offspring environments, producing 12 different treatment combinations (Fig. 1). We also tracked parental care intensity (i.e., the average proportional time spent with the clutch) over the 4-day long care period in the four treatment combinations that involved parental care as a possible mechanism of transgenerational risk transmission. As we also consistently manipulated clutch size, either by removing some eggs to raise them in the absence of parental care, or by swapping clutches between parents for cross-fostering treatments, we also assessed to what extent the resulting proportional change in clutch size impacted parental care intensity.

Shoaling is a form of animal grouping well-known for its antipredator function in reducing risk for individual shoaling members [51, 52]. While the formation of dense shoals can additionally be beneficial in the context of social foraging, it is also associated with greater resource competition as well as higher disease transmission risk [53, 54]. Resulting from a trade-off between these costs and benefits, shoaling density increases as a function of the level of acute predation risk [54], which makes this trait well-suited to investigate risk assessment. That is why we studied shoaling density in 39-day old offspring. Furthermore, we studied the change in shoaling density following a novel predator disturbance, as it is established as another reliable method to reveal risk assessment in fish [48, 55]. Following the risk allocation hypothesis, which postulates lower investment into antipredator strategies under consistent high-risk so as to satisfy metabolic requirements [56, 57], *P. promelas* raised under continuous high background risk resume normal shoaling densities quicker after a novel disturbance [48]. In contrast, low-risk fish form denser shoals for a longer time after exposure to a novel stimulus [48]. Consequently, in our study we expect shoals that estimate risk to be high from either parental or personal information to form denser shoals during the prestimulus period and to show risk allocation, i.e., to quickly return to prestimulus shoaling densities. In contrast, shoals that estimate risk to be low should form loose shoals during the prestimulus period and denser shoals following the stimulus. We also consider the possible influence of other factors that may impact plastic responses such as maternal [58] or paternal condition [59] as well as other factors that may impact shoaling density such as the size of individuals within shoals as well as shoal homogeneity (i.e., variance in size within shoals) [details in 55].

Given that previous studies suggest transmission of risk through maternal gametes, paternal gametes and parental care, we predict that high-risk experienced by the mother, by the father and by the caring individuals all impact offspring risk assessment. Furthermore, given that gamete-mediated effects are generally weak across multiple meta-analyses [60, 61] and that Steiger [27] suggests parental care effects to far outweigh prenatal effects, our hypothesis is that the impact of the parental care environment will far outweigh the impact of gamete-mediated maternal and paternal effects alone. Additionally, following previous research on the impact of immediate risk of parental care intensity [22, 62, 63], we expect that high-risk caring parents that were lifelong exposed to high-risk prior to parental care will likewise provide less care to offspring. Furthermore, as parental care intensity has been shown to directly modulate antipredator responses in offspring [23, 24], we predict that parental care intensity may be a major mechanism that transmits risk across generations. Lastly, we expect that parentally transmitted information is integrated with the offspring environment, with the offspring environment having a greater impact on phenotypes as it is a more accurate predictor of future environmental conditions [8].

## Results

### Paternal care intensity

Three factors shaped variation in parental care intensity (Table 1). Paternal care intensity was influenced by the day of care (R^2^ = 0.020, 95% CI of R^2^: [0, 0.230], F_1,31.733_ =  6.035, p = 0.020) with ca. 10% increases in care over the 4-day period. Additionally, the proportional change in clutch size modified parental care intensity (R^2^ = 0.064 [0, 0.258], F_1,39.334_ = 5.730, p = 0.022). Males provided less care the more eggs we removed for our split-clutch “absence of parental care” treatments, and more care when the clutches that they received during cross-fostering contained more eggs, leading to up to ca. 40% differences in care intensity. Beyond these effects, paternal care intensity was best explained by whether the father provided care to its own clutch or to an adopted clutch (R^2^ = 0.132 [0.012, 0.316], F_1,25.723_ = 13.137, p = 0.001) rather than by risk treatment (see Additional file 1: Section 2). Offspring from clutches that received parental care from their original fathers (i.e., the biparental low-risk treatment that received care by a low-risk male and the biparental high-risk treatment that received care by a high-risk male) received ca. 12.4% more care than did offspring from treatments where clutches were adopted.

**Table 1:** Full and final linear mixed-effect models analysing variation in *Pimephales promelas* parental care intensity

	df_Numerator_ for fixed effects	df_Denominator_ for fixed effects	F for fixed effects, χ^2^ for random effects	*P*
**Variation in parental care**				
*Full model *				
Care type × day of care	1	58.930	0.276	0.602
Care type × final clutch size	1	40.541	0.487	0.489
Care type × proportional change in clutch size	1	41.487	1.184	0.283
Day of care	1	29.439	6.198	0.019
Final clutch size	1	39.478	0.731	0.398
Proportional change in clutch size	1	41.589	1.755	0.193
Care type	1	39.905	2.936	0.094
Caring parent ID × family			4.351	0.114
Family			1.616	0.446
*Final model*				
Day of care	1	31.735	6.035	0.020
Proportional change in clutch size	1	39.337	5.731	0.022
Care type	1	25.724	13.138	0.001
Caring parent ID × family			5.114	0.078
Family			1.498	0.473

Care type (i.e., taking care of own or adopted eggs) was the fixed effect of interest. Variation in parental care (i.e., proportion of time spent next to the clutch) was Yeo-Johnson transformed before analysis

### Offspring morphology

Neither variation in average body size of shoals nor differences in within-shoal variation in body size (i.e., shoal homogeneity) were explained by any of the fixed effects or their interactions with risk treatment (all p ≥ 0.078, Additional file 1: Table S2).

### Prestimulus shoaling density

**Fig. 2:**
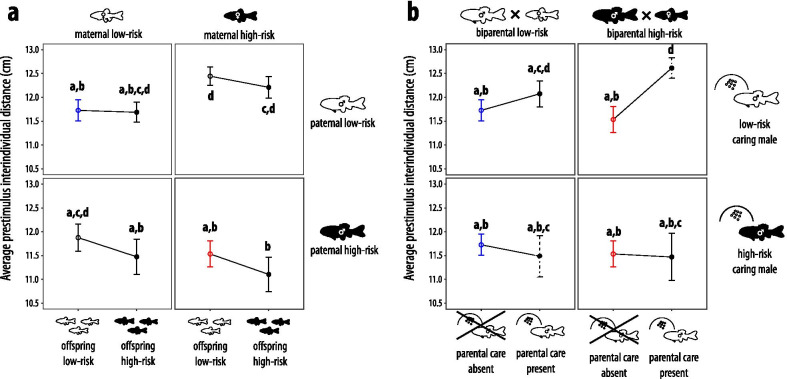
Average (over a 10-min period) prestimulus interindividual distances (mean ± SE) of 39-day old fathead minnows showcasing (**a**) maternal × paternal × offspring risk interactions and (**b**) biparental × caring parent × presence of parental care risk interactions. In **a**, white dots represent offspring low-risk whereas black dots represent offspring high-risk environments. In **b**, offspring were always exposed to low-risk environments only; hence in **b** white dots represent the absence of parental care whereas black dots represent the presence of parental care. Within fish drawings, black fish indicate individuals that were from birth onwards exposed to conspecific alarm cues, white fish refer to the ones that instead received a water control treatment; for more detail see Fig. 1. Black circles and error bars represent treatments shown only once across figures, for every other color, same-colored markers refer to the same treatment across figures. Dashed error bars in **b** highlight the treatments that were cross-fostered. Different letters above and below bars indicate statistical differences at p < 0.05 according to post-hoc tests from linear mixed-effect models that also contained all remaining covariates (final model in Table 2)

Three factors explained variation in prestimulus shoaling density (Table 2). Shoals with larger average body sizes (R^2^ = 0.016, 95% CI of R^2^: [0, 0.160], F_1272.434_ = 5.012, p = 0.026) and fish in heterogeneous shoals (R^2^ = 0.036 [0.006, 0.180], F_1279.452_ = 10.990, p = 0.001) generally formed less dense shoals independent of the treatment (Table 2). In addition, risk treatment influenced shoaling density (R^2^ = 0.071 [0.044, 0.214], F_11,128.894_ = 2.270, p = 0.014, Fig. 2). Overall, largest average IIDs (i.e., least dense shoals) during the prestimulus period were observable in offspring that originated from a biparental high-risk environment and were cared for by low-risk males (d_Cohen_ = 0.607, 95% CI of d_Cohen_: [0.148, 1.067], F_1,278_ = 5.766, p = 0.017). Within biparental high-risk shoals, low-risk caring males induced greater IIDs than high-risk caring males (d_Cohen_ = 0.692 [0.023, 1.360], t_259.1_ =  2.675, p = 0.008), and also greater ones than when the same offspring did not receive any parental care (d_Cohen_ = 0.690 [0.135, 1.245], t_252.4_ =  3.317, p = 0.001). Shortest average IIDs (i.e., the densest shoals) were observable in one of the no-care treatments, specifically in the one where offspring from high-risk parents were themselves exposed to high-risk (d_Cohen_= 0.492 [0.044, 0.941], F_1278_ = 8.238, p = 0.004). This suggests additive effects of exposure to high-risk in both the parental and the offspring generation. Additionally, in the absence of care, paternal high-risk alone induced shoaling densities that were not significantly different from biparental high-risk (personal low-risk: d_Cohen_ =  0.209 [−0.324, 0.742], t_267_ = 0.920, p = 0.359; personal high-risk: d_Cohen_ = 0.228 [−0.415, 0.871], t_267_ = 1.376, p = 0.170). This was not the case for maternal high-risk, which induced less dense shoals than biparental high-risk treatments (personal low-risk: d_Cohen _ = 0.585 [0.024, 1.146], t_267_ = 2.632, p = 0.009, personal high-risk: d_Cohen_ = 0.808 [0.167, 1.145], t_267_ = 3.431, p < 0.001). Personal risk had no significant effect on prestimulus shoaling densities (Fig. 2). Moreover, variation in parental care intensity was not related to variation in prestimulus shoaling density (Table 3).

**Table 2:** Full and final linear mixed-effect models analysing variation in *Pimephales promelas* shoaling density

	df_Numerator_ for fixed effects	df_Denominator_ for fixed effects	F for fixed effects, χ^2^ for random effects	*P*
**Prestimulus shoaling density**				
*Full model *				
Risk treatment × paternal condition	11	148.372	0.772	0.667
Risk treatment × maternal condition	11	140.796	1.268	0.249
Risk treatment × average size in shoal	11	264.480	1.481	0.138
Risk treatment × shoal homogeneity	11	264.351	1.373	0.185
Paternal condition	1	50.328	4.988	0.030
Maternal condition	1	45.140	0.000	0.988
Average size in shoal	1	254.976	8.358	0.004
Shoal homogeneity	1	280.986	11.717	< 0.001
Risk treatment	11	195.375	1.595	0.103
Family			42.265	< 0.001
*Final model *				
Average size in shoal	1	272.430	5.012	0.026
Shoal homogeneity	1	279.450	10.990	0.001
Risk treatment	11	128.890	2.270	0.014
Family			63.544	< 0.001
**Change in shoaling density **				
*Full model *				
Risk treatment × paternal condition	11	281	1.447	0.152
Risk treatment × maternal condition	11	281	1.729	0.067
Risk treatment × average size in shoal	11	281	1.409	0.168
Risk treatment × shoal homogeneity	11	281	0.481	0.915
Paternal condition	1	281	0.079	0.779
Maternal condition	1	281	2.513	0.114
Average size in shoal	1	281	3.589	0.059
Shoal homogeneity	1	281	13.711	< 0.001
Risk treatment	11	281	1.722	0.068
Family			< 0.001	1.000
* Final model*				
Shoal homogeneity	1	281	9.033	0.003
Risk treatment	11	281	2.911	0.001
Family			< 0.001	1.000

**Table 3:** Full and final linear mixed-effect models analysing parental care-related factors on *Pimephales promelas* shoaling density

	df_Numerator_ for fixed effects	df_Denominator_ for fixed effects	F for fixed effects, χ^2^ for random effects	*P*
**Prestimulus shoaling density**				
*Full model *				
Average care intensity × risk treatment	2	17.540	1.664	0.218
Slope of care intensity × risk treatment	2	13.633	0.672	0.527
Average care intensity × care type	1	14.938	4.463	0.052
Slope of care intensity × care type	1	21.839	0.485	0.494
Average care intensity	1	14.381	3.393	0.086
Slope of care intensity	1	18.498	0.094	0.763
Risk treatment	2	18.630	1.481	0.253
Care type	1	15.871	4.301	0.055
Caring parent ID × family			0.000	0.990
Family			1.863	0.172
*Final model*				
Caring parent ID × family			6.168	0.013
Family			0.532	0.466
**Change in shoaling density **				
*Full model *				
Average care intensity × risk treatment	2	75	1.939	0.151
Slope of care intensity × risk treatment	2	75	3.713	0.029
Average care intensity × care type	1	75	0.161	0.690
Slope of care intensity × care type	1	75	0.810	0.371
Average care intensity	1	75	1.831	0.180
Slope of care intensity	1	75	4.654	0.034
Risk treatment	2	75	2.270	0.110
Care type	1	75	0.066	0.798
Caring parent ID × family			< 0.001	1.000
Family			< 0.001	1.000
*Final model *				
Average care intensity × care type	1	56.635	10.353	0.002
Average care intensity	1	73.983	14.616	< 0.001
Risk treatment	2	37.101	5.841	0.006
Care type	1	57.968	9.439	0.003
Caring parent ID × family			< 0.001	1.000
Family			1.694	0.193
*Final model—own offspring*				
Average care intensity	1	37	13.600	< 0.001
Risk treatment	1	37	8.423	0.006
Caring parent ID × family			< 0.001	1.000
Family			< 0.001	1.000
*Final model—adopted offspring*				
Average care intensity	1	18.552	0.235	0.633
Risk treatment	1	18.516	0.721	0.407
Caring parent ID × family			< 0.001	1.000
Family			< 0.001	1.000

Only the data from the four parental care treatments are analyzed here

### Change in shoaling density

**Fig. 3:**
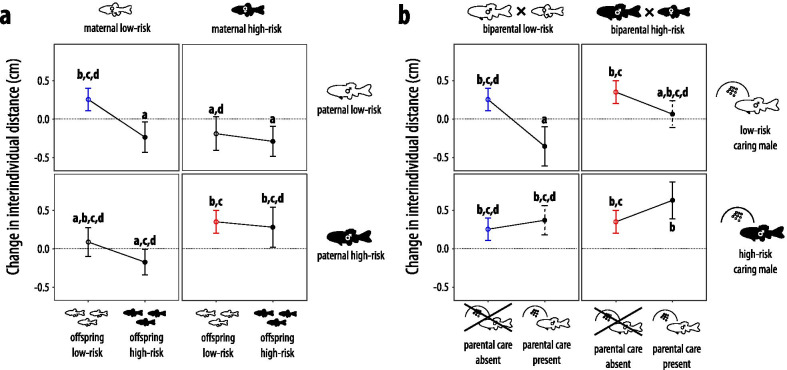
Average change in shoaling densities (mean ± SE) of 39-day old fathead minnows as induced by a mechanical predator disturbance showcasing (**a**) maternal × paternal × offspring risk interactions and (**b**) biparental × caring parent × presence of parental care risk interactions. The dashed line is the zero referent and represents no change in shoaling density. In **a**, empty dots represent offspring low-risk whereas filled dots represent offspring high-risk environments. In **b**, offspring were always exposed to low-risk environments only; hence empty dots in **b** represent the absence of parental care whereas filled dots represent the presence of parental care. Within fish drawings, black fish indicate individuals that were from birth onwards exposed to conspecific alarm cues, white fish refer to the ones that instead received a water control treatment; for more detail see Fig. 1. Black circles and error bars represent treatments shown only once across figures, for every other color, same-colored markers refer to the same treatment across figures. Dashed error bars in **b** highlight the treatments that were cross-fostered. Different letters above bars indicate statistical differences at p < 0.05 according to post-hoc tests from linear mixed-effect models that also contained all remaining covariates (final model in Table 2). Raw values are plotted in Additional file 1: Fig. S1

Variation in the change in shoaling density was best explained by two factors (Table 2). First, heterogeneous shoals generally reduced offspring shoaling density following the stimulus to a greater extent (R^2^ = 0.029, 95% CI of R^2^: [0, 0.145], F_1281_ = 9.033, p = 0.003). Otherwise only the risk treatment modulated variation in the shoaling density changes (R^2^ = 0.101 [0.072, 0.218], F_11281_ = 2.911, p = 0.001, Table 2; Fig. 3, Additional file 1: Fig. S1). During model reduction, we had also discovered a significant risk treatment × average size within shoals interaction, but further analysis revealed this interaction to be inconsequential (see Additional file 1: Section 3).

Overall, largest average increases in IIDs (i.e., patterns of risk allocation) were observable in offspring that originated from a biparental high-risk environment and were cared for by high-risk males (d_Cohen_ =  0.591, 95% CI of d_Cohen_: [0.121, 1.061], F_1279_ = 6.114, χ^2^ = 5.914, p = 0.014), suggesting additive effects of parental and caring parent risk (Fig. 3, Additional file 1: Fig. S1). Parental care by high-risk males induced similar levels of risk allocation in biparental low-risk offspring as in biparental high-risk offspring (d_Cohen_ = 0.278 [−0.393, 0.949], t_268_ = 0.846, p = 0.399). At the same time, without any other sources of risk information involved, parental care by high-risk males clearly induced greater levels of risk allocation than low-risk care (d_Cohen_ = 0.757 [0.056, 1.458], t_284_ = 2.563, p = 0.011), than maternal high-risk (high personal risk: d_Cohen_ =  0.763 [0.097, 1.429], t_268_ = −2.386, p = 0.018; low personal risk: d_Cohen_ =  0.634 [−0.050, 1.318], t_268_ = 1.923, p = 0.056) and than personal high-risk (d_Cohen_ = 0.707 [0.036, 1.378], t_269_ = −2.051, p = 0.041).

**Fig. 4:**
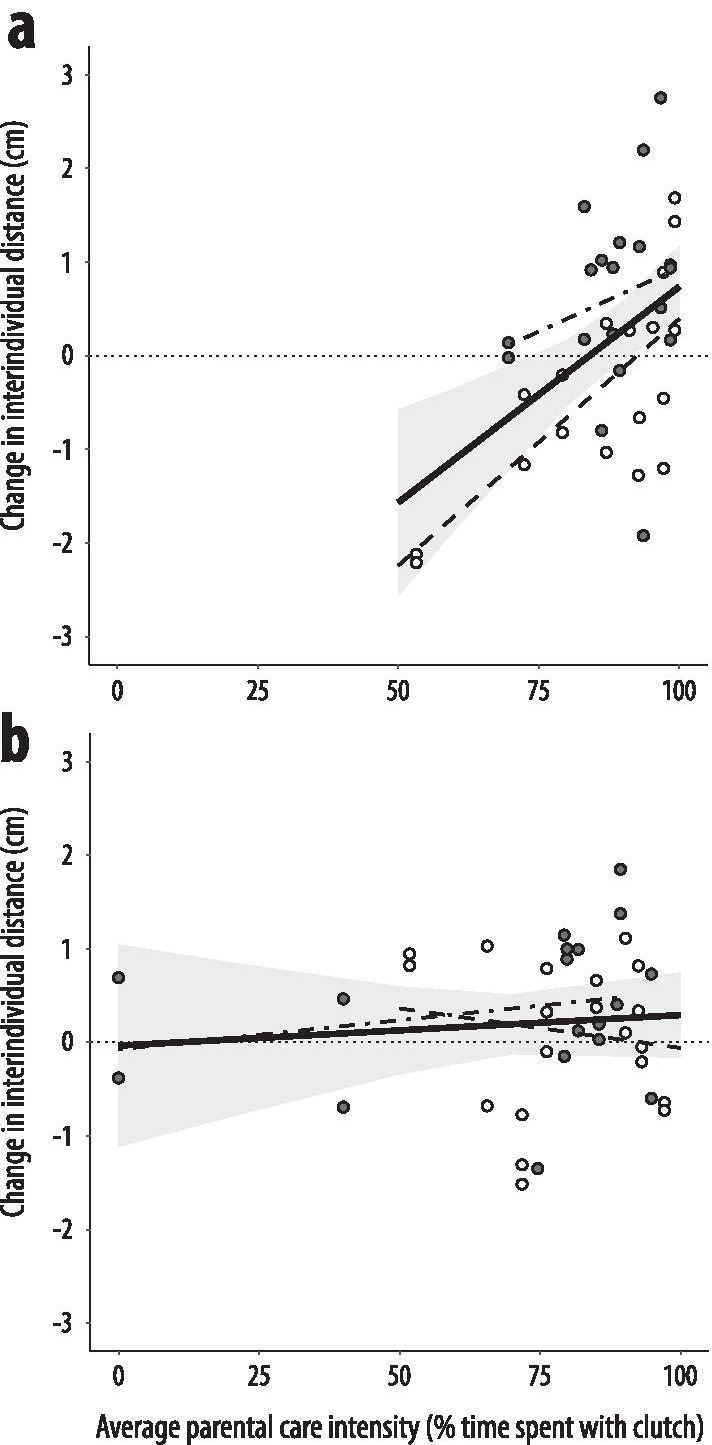
Relationship between average paternal care intensity and the change in interindividual density following the mechanical predator disturbance for (**a**) own and (**b**) adopted offspring from the opposing risk treatment, respectively. Filled dots visualize instances where high-risk males provided care, empty dots instead refer to care by low-risk males. Shaded areas (95% confidence intervals) and regression lines for the treatment-independent correlation (thick solid line) were estimated from mixed-effect models with treatment (low-risk/high-risk) as covariate and caring individual ID nested in shoal family origin as random intercept. Regression lines for treatment-specific correlations (low-risk care: dashed lines, high-risk care: dot-dashed lines) are shown without confidence intervals to avoid visual clutter. The dotted line is the zero referent and represents no change in shoaling density

Largest decreases in IIDs as induced by the stimulus were observable in offspring from a biparental low-risk environment that were cared for by low-risk males (d_Cohen_= 0.504 [0.023, 0.985], F_1,279_ =  5.759, p = 0.017). Further, the two care types (taking care of own or adopted eggs) differed in the extent that average parental care intensity impacted on the change in shoaling density (Table 3); when own eggs were cared for, both higher care intensities (R^2^ = 0.227 [0, 0.474], F_1,37_ = 13.600, p < 0.001, Fig. 4a) and high-risk experience in the caring male (R^2^ = 0.138 [0, 0.400], F_1,37_ = 8.423, p = 0.006) induced greater levels of risk allocation, but this effect was absent when eggs were adopted (care intensity: R^2^ = 0.009 [0, 0.321], F_1,18.552_ = 0.235, p = 0.633; risk treatment: R^2^= 0.027 [0, 0.347], F_1,18.516_ =  0.721, p = 0.407, Fig. 4b).

In the absence of parental care, biparental high-risk induced greater risk allocation than offspring from low-risk parents that experienced personal high-risk (personal low-risk: d_Cohen_ = 0.611 [0.068, 1.154], t_268_ = 2.443, p = 0.015; personal high-risk: d_Cohen_ = 0.489 [−0.144, 1.122], t_268_ = 2.068, p = 0.040) as well as than maternal-only high-risk exposure (personal low-risk: d_Cohen_ = 0.553 [−0.007, 1.113], t_268_ = 2.263, p = 0.025; personal high-risk: d_Cohen_ = 0.540 [−0.087, 1.167], t_268_ = 2.405, p = 0.017). Furthermore, paternal high-risk alone again generated offspring with similar patterns of risk allocation to biparental high-risk (personal high-risk: d_Cohen_ = 0.454 [−0.196, 1.103], t_268_ = 1.624, p = 0.106; personal low-risk: d_Cohen_ =  0.277 [−0.257, 0.811], t_268_ = 0.846, p = 0.399). Maternal high-risk alone induced intermediate responses and consequently differed from biparental high-risk (all p ≤ 0.025) but not from either biparental low-risk (all p ≥ 0.054) or paternal high-risk (all p ≥ 0.228). Different offspring environments impacted shoaling density only within the biparental low-risk treatment (d_Cohen_ = 0.534 [−0.014, 1.082], t_265_ = 2.101, p = 0.037). Here, high-risk offspring reduced their IIDs in response to the mechanical stimulus whereas low-risk offspring increased them. This suggests that personal environments have a lower impact when high-risk is present in the parental generation (Fig. 3, Additional file 1: Fig. S1).

## Discussion

Across risk treatments, the involvement of parental care consistently induced both highest and lowest shoaling density responses. Prior to any stimulus, offspring from high-risk parents that received care by low-risk males formed the least dense shoals, which is a typical low-risk response (Fig. 2). Furthermore, additive effects from the same biparental and parental care risk levels induced stronger responses that follow the predictions by the risk allocation hypothesis: a quick return to prestimulus shoaling densities after a novel mechanical stimulus in individuals from high-risk treatment combinations but not in low-risk ones (Fig. 3). Paternal care intensity was the highest if the offspring they cared for was their own, and care intensity showed a weak positive correlation with clutch age as well as with the proportional change in clutch size due to experimental manipulation (Table 1). Furthermore, care intensity was positively correlated with the level of risk allocation when parents took care of their own eggs (Fig. 4). In the absence of parental care, maternal effects, which were subtle to non-existent, were outweighed by paternal effects, but biparental high-risk induced the densest shoals and the greatest extent of risk allocation (Figs. 2 and 3). The impact of personal risk environments was generally low and only statistically significant when no risk was present in the parental generation (Figs. 2 and 3). Generally, the present study highlights the high importance of parental care relative to gamete-mediated effects in shaping offspring shoaling density and risk allocation patterns.

Treatments involving parental care were consistently the ones that induced greatest shoaling density responses and these outweighed gamete-induced effects for both prestimulus density and the change in shoaling density (Figs. 2 and 3). This is in accordance with multiple meta-analyses that question the adaptiveness of gamete-mediated maternal and paternal effects alone [60, 61] and with the suggestion that effects of parental care outweigh prenatal effects [27]. The general direction of parental care risk environments (i.e., while not always being statistically significant, high-risk care induced at least slightly denser shoals and risk allocation whereas low-risk care induced the opposite pattern; Figs. 2b and 3b) appears to support the hypothesis that prenatal high-risk and low-risk in the caring individuals induces putatively adaptive responses typical for high and low-risk, respectively. While a larger sample size may be necessary to reveal a significant impact of these effects across all risk treatments that involved parental care, a similar pattern was previously observed in response to high and low immediate risk during care [23].

Variation in parental care intensity was to a small extent explained by whether eggs were adopted or not, proportional changes in clutch size and the age of the eggs (Table 1, see Additional file 1: Section 4 for discussion of these effects). However, even when statistically controlling for these slightly confounding factors, there was no general effect of prenatal caring parent risk on parental care intensity (Table 1), contrasting other studies reporting that immediate high-risk environments induce lower parental care intensities [22, 62]. This lower than expected impact of prenatal risk environments on parental care intensity may result from us using a *P. promelas* population that has been kept in the laboratory dating back to 1985, and possibly earlier (see Additional file 1: Section 1 for more detail). Such laboratory populations may not have evolved predator-induced plasticity in parental care intensity due to the absence of fluctuating risk [sensu 64]. Consequently, risk-induced differences in parental care intensity are unlikely to be the main mechanism for the transmission of risk information across generations. Instead, because fish embryos are capable of detecting environmental cues from within their eggs across taxa [65, 66], caring males may transfer prenatal risk to offspring through mechanisms that are not correlated with care intensity such as the release of disturbance cues [67], steroid hormones [68] or mechanosensory cues [69] following the theory that adult behaviours may mimic environmental cues experienced by the parents themselves [70].

In addition, when fathers took care of their own offspring, independent of prenatal risk levels, high levels of paternal care intensity also induced greater offspring risk allocation (Fig. 4a). This result matches previous observations across fish taxa that parental care intensity directly modulates antipredator responses in offspring [23, 24]. The proximate mechanism here may be glucocorticoid receptor methylation differences that are associated with variation in parental care intensity [71, 72]. As perceived predation risk elevates glucocorticoid levels in prey [73, 74], care-induced differences in associated receptor expression are likely to affect offspring responses to perceived predation risk. That care intensity was not correlated with offspring risk allocation in cross-fostered clutches (Fig. 4b) may either be a consequence of environmental mismatches between parental and parental care environment or alternatively a by-product of cross-fostering negatively impacting parental care intensities. Future studies are clearly required to disentangle these two possibilities.

Within treatments that did not involve parental care, paternal risk effects mostly outweighed maternal effects in terms of the adaptiveness of the response for a high-risk environment for both prestimulus shoaling density and the change in shoaling density (Figs. 2a and 3a), suggesting the presence of sex-specific transgenerational plasticity [21]. The general direction of paternal effects mostly outweighing maternal effects contrasts other studies across taxa suggesting that usually maternal effects outweigh paternal effects [snails: 75, birds: 76, fish: 77]. Our observation is still in accordance with many studies highlighting the relevance of paternal effects [78, 79], and a recent meta-analysis suggesting that paternal effect sizes are on average larger than maternal ones [61]. This could be because paternal sperm methylomes are known to be inherited to offspring unaltered whereas maternal methylation patterns experience substantial reorganization [80, 81]. Alternatively, paternal environmental information may be more reliable than maternal information [21], mainly because caring male *P. promelas* are more likely to share their environment with offspring compared to females which disperse after egg deposition. However, we cannot exclude the possibility that the few minutes to hours that fathers took care of the embryos immediately after egg deposition might already be sufficient to transmit some information about the paternal environment via parental care, making the presumably paternal gamete-mediated effects possible by-products of paternal care effects. Furthermore, paternal high-risk did not induce different effects from personal experience with high-risk (Figs. 2a and 3a), in accordance with a previous stickleback study [82]. In the absence of care, biparental high-risk consistently induced the on average densest shoals (Fig. 2a) and the on average greatest extent of risk allocation (Fig. 3a), suggesting that maternal and paternal perceived risk have additive effects on offspring, in accordance with some previous stickleback studies [83] but contrasting other stickleback research suggesting non-additive effects [16, 84]. These additive effects between maternal and paternal environments are then further additively exacerbated by high-risk parental care (Figs. 2b and 3b), which, following the theory of co-adaptation of prenatal and postnatal effects [26], helps to maximize risk allocation in high-risk environments.

Parental environments sometimes also affected the impact of personal environments on shoaling density. Although during the prestimulus period, high personal risk appears to slightly increase shoaling density mostly independent of parental risk levels (Fig. 2a), which is an adaptive response [51, 52], these effects were not statistically significant. The only significant effect of high personal risk emerged following the stimulus (Fig. 3a), matching the finding that some level of immediate risk is required to reveal the effects of background risk exposure [48, 75]. High personal risk appears to reduce risk allocation to a greater extent the less information about high-risk is present in the parental generation, culminating in a significantly reduced risk allocation only when both parents were derived from a low-risk environment (Fig. 3a). This result is in accordance with information theory that predicts decreasing confidence in information when different sources provide conflicting cues [85]. Accordingly, mismatches between parental and personal risk communicate that background risk levels are not consistent, an instance where risk allocation is not adaptive. Compared to personal risk, parental risk levels were more important in modulating risk allocation. This result is in accordance with Bayesian updating theory, which predicts a greater reliance on parental risk information especially during juvenile life-stages, as parents have had the opportunity to repeatedly sample the environment over longer temporal scales [86]. By relying more on parental information, juveniles can effectively avoid the danger of overestimating spurious fluctuations in risk, especially in species such as *P. promelas* where predator presence is putatively highly predictable over multiple generations. Furthermore, that we did not observe significantly better-adapted phenotypes when both parental and personal risk were high (Figs. 2a and 3a) is in accordance with the reported benefits of environmental matching across generations being weak in general [60].

Lastly, independent of treatment, large average fish size within shoals and heterogeneous shoals had small negative effects on shoal densities; this result is largely independent of measuring these effects in absolute or relative units (Additional file 1: Table S3). This follows the theory that predation risk is higher for smaller fish [87], and that smaller fish should avoid shoaling with larger conspecifics due to food competition [88].

## Conclusions

Taken together, our study highlights for the first time the high relative importance of risk transmission through parental care as opposed to other sources of risk information within and across generations. Furthermore, the widespread neglect of parental care effects during transgenerational research may thus contribute to small observed effect sizes [60, 61]. Consequently, we hope that our study encourages more research on the exact mechanism for risk transmission during parental care and a greater focus on parental care as a mechanism underlying presumably inherited gene regulation.

## Methods

### Experimental fish

In collaboration with the University of Saskatchewan’s Aquatic Toxicology Research Facility (ATRF), Canada, we bred adult *P. promelas* sourced from their own predator-free laboratory stock population to generate the parental fish for our experiment [47–49]. Eggs were collected after deposition, moved to the University of Saskatchewan’s RJF Center for Aquatic Ecology and raised using a split-clutch design based on exposing individuals continuously from birth onwards to either conspecific alarm cues [which reliably signals high-risk and induces typical antipredator phenotypes across taxa, including fathead minnows, see 48, 67, 89] or a distilled water control (low-risk). In contrast to predator cues, alarm cues are innately recognized [90] and no habituation occurs even after repeated exposure [91]. As the fish in the ATRF were sexually mature, which reduces alarm cue efficacy [92, 93], alarm cue donors were non-reproductive conspecifics collected from the University of Saskatchewan’s Feedlot Pond (52°09’23.4"N, 106°37’04.5"W). Alarm cues from this population have been proven multiple times to be recognizable to conspecifics as high-risk cues [45, 47–49, 94]. In the next generation, we used our parental individuals to generate a total of 12 treatment combinations using a similar set-up (Fig. 1). First, in the absence of parental care, risk levels were crossed across maternal, paternal and offspring environments in a full-factorial 2 × 2 × 2 design. Additionally, to investigate the relative importance of parental care, we further split some of the previously used clutches so as to generate a 2 × 2 factorial design that crossed (genetic) biparental risk with caring parent risk; offspring consistently grew up in a low-risk environment here. In the two treatments where caring parent risk was mismatched with biparental risk levels, we cross-fostered offspring; although we intended to do so for all four parental care treatments, the number of pairs from the same parental environment combination that spawned concurrently was insufficient. At the same time, space limitations did not allow us to investigate effects of parental care for other maternal, parental and offspring risk environment combinations. For additional details of the raising process see Additional file 1: Section 1.

### Parental care

To assess variation in parental care intensity in the four treatments that involved parental care, we recorded caring male behaviour every day until hatching (4 days) for a period of 10 min between 1600 and 1900 h, before feeding, using a camera (C922x Pro Stream, Logitech, Suzhou, China).

### Shoaling assays

We used 39-day old fish to run the shoaling assays as described in Meuthen et al. [48]. In brief, 21.4 × 21.4 × 21.8 cm white pails are filled with 500 ml water (temperature 20.0 ± 0.1 °C), resulting in a shallow water depth of 1.09 cm, which allows evaluating between-fish distances in two dimensions [95] and at the same time represents a naturally realistic context for shoals of juvenile *P. promelas* as they typically inhabit shallow water habitats [40]. A predator disturbance stimulus could be provided through a wooden apparatus that provided a hit of 0.108 joules to the bucket [48]; this novel mechanical stimulus reliably induces typical antipredator responses [48, 55]. Trials were video-taped (C922x Pro Stream). Experimental shoals were gently introduced in the set-up, this was followed by a 10-min acclimation period. Afterwards, the experiment started with a 10-min prestimulus period, followed by the delivery of the mechanical predator disturbance stimulus, and a 10 min poststimulus period. In total, we tested 281 shoals of 10 fish each (see Fig. 1 for details). We had originally tested 10 additional shoals but had to exclude them due to issues with nearby construction noise [which affects antipredator responses, see 96], power outages, unexpected mortality immediately before the trial, or procedural errors by the experimenter.

### Data analysis

#### Parental phenotypes

As maternal [58] or paternal condition [59] may influence antipredator plasticity, for every parental individual, we calculated condition indices according to Bolger and Connolly [97]: [100 * mass (g)]/standard length (cm)^x^, with x = 3.043 as assessed by regressing log_10_ weight against log_10_ standard length over all parental individuals. Neither maternal (Wilcoxon rank-sum test, W = 222.5, p = 0.754) nor paternal condition (Wilcoxon rank-sum test, W = 214.5, p = 0.705) differed between parental risk treatments.

#### Parental care

To standardize the time from moving the camera in front of the tank (i.e., a visual disturbance), we analyzed only the last 5 min of each recording, an established time period across minnow parental care studies [98]. We found that male egg manipulation such as rubbing and mouthing occurs almost constantly when males were in the vicinity of the clutch (often more than once per second, preventing repeatability when counting), hence in every recording we measured the relative time the male spent within one standard length of the eggs (inside the breeding tile) as is done as a typical measure of parental care intensity across taxa [99]. Four recordings per clutch were analyzed, in total 160 videos from 20 different caretakers that cared for 40 clutches. For every clutch, we calculated the average relative time that the male spent with the clutch as a proxy for average parental care intensity. Furthermore, as variation in parental investment between treatments may reflect in the slope of parental care over time [99], for each clutch we performed robust regression by calculating Theil-Sen-Siegel slopes of the care given over the 4-day period; using such repeated medians to estimate slopes is robust to outliers [100].

#### Size measurements

To assess the size of fish within shoals, we selected a frame from each video where fish movement was minimal and where fish bodies were as straight as possible. We extracted this frame using VirtualDub (A. Lee 1998–2012; version 1.10.4) and measured fish sizes (total length, i.e., from the tip of the snout to the end of the tail fin) in ImageJ (Rasband 1997–2018, US National Institutes of Health, Bethesda, MD, USA). By using known pail size as a reference, we converted the fish size in pixels into millimeters. For each shoal, we first calculated the average body size. Then, as a proxy for shoal homogeneity, which is known to impact shoaling behavior [55, 101], we also calculated coefficients of variation for each shoal (CV, dividing the standard deviation by the mean).

#### Shoaling distance measurements

To assess shoaling distances, we extracted 1 image every 30 s from the video file, resulting in 40 images per shoal (20 pre- and 20 post-stimulus images). We then measured interindividual distances (IIDs), which are more precise than nearest-neighbor distances when shoal size is kept constant across trials [102] using tpsDig2 2.30 (F. James Rohlf, Stony Brook University, USA) and CoordGen 8 (Integrated Morphometrics Package Suite, H. David Sheets, Canisius College, USA). As 10 fish were present in each image, 45 IIDs could be extracted from every frame. In total, from 281 shoals of 10 fish each (18–43 shoals per risk treatment, see Fig. 1), we generated 11,240 images and measured 505,800 IIDs. First, we averaged IIDs for each frame, and then for each shoal, we calculated mean IIDs over the 10 min prestimulus period and the following 10 min poststimulus period. Within-shoal variation in IIDs over frames was small and did not differ between treatments (mean ± SD coefficient of variation along with Kruskal-Wallis tests; overall 0.215 ± 0.060, χ^2^ = 9.501, df = 11, p = 0.576; prestimulus 0.211 ± 0.065, χ^2^ = 8.443, df = 11, p = 0.673; poststimulus 0.214 ± 0.065, χ^2^ = 13.331, df = 11, p = 0.272). Afterwards, we also calculated the average change in IID that was induced by the mechanical stimulus (post-pre). Assessing the change in IID as proportional change from the prestimulus baseline (i.e., post-pre/pre) in preliminary analyses did not qualitatively or quantitatively affect our results. All data from this study are deposited in Additional file 2.

### Statistical analysis

All analyses were conducted with R 4.0.3 [103], accompanied by the packages lme4 1.1-26 [104], lmerTest 3.1-3 [105], emmeans 1.5.4 [106], effsize 0.8.1 [107] and partR2 0.9.1 [108]. We applied linear mixed-effect models with family identity as a random intercept using maximum likelihood parameter estimation throughout; full models were then subject to stepwise reduction using backward elimination procedures with Satterthwaite approximations (retaining random factors) until only significant fixed effects remained in final models. Quantile-quantile plots of model residuals were inspected to ascertain that parametric assumptions were satisfied. In the final models, we calculated partial R² values and Cohen’s d along with 95% confidence intervals to visualize effect sizes. First, we analyzed whether risk treatment, care type (own/adopted), the day of care, final clutch size (i.e., the number of eggs that were cared for), the change in clutch size or any of their interactions with risk treatment explained variation in parental care intensity (Yeo-Johnson transformed), with day of care as a random slope and caring male ID as nested random intercept. Afterwards, we aimed to reveal whether risk treatment, original clutch size (i.e., the total number of eggs in the clutch they were derived from), parental condition or their interactions with risk treatment explained variation in the average size within shoals or within-shoal variation in body size (i.e., shoal homogeneity). Third, we assessed whether risk treatment, average size within shoals, shoal homogeneity, parental condition or any of their interactions with risk treatment explained variation in prestimulus IID or the change in IID. Fourth, for the four treatments that involved parental care, we assessed to what degree variation in parental care intensity or in the slope of parental care explained variation in both prestimulus IID or the change in IID (with caring male ID as nested random intercept). When risk treatment effects were significant in the final models, we conducted pairwise post-hoc analyses. Families were not shared among all treatment combinations, hence we present the results from post-hoc analyses with family identity as a random effect only where it is appropriate (in 14 of 66 pairwise comparisons); otherwise, we provide data from post-hoc models without random effects.

## Supplementary Information

**Additional file 1.** **Section 1.** Breeding, rearing and alarm cue exposure protocol. **Section 2.** Supporting results: Variation in parental care intensity. **Section 3.** Supporting results: Variation in the change in shoaling density. **Section 4.** Supporting discussion: Other factors affecting parental care intensity. **Table S1.** Full and final linear mixed-effect models analysing the factors explaining variation in Pimephales promelas parental care intensity, with risk treatment as the fixed effect of interest. **Table S2.** Full and final linear mixed-effect models analysing the factors explaining variation in average body size within shoals and within-shoal variation in body size, respectively. **Table S3.** Impact of average fish size within shoals and shoal heterogeneity on prestimulus shoaling density and the change in shoaling density when density is measured in **a** absolute distances, i.e., centimeters and **b** relative distances, i.e., # of body lengths. **Fig. S1.** Average (over a 10-min period) interindividual distances (mean ± SE) of 39-day old fathead minnows (Pimephales promelas) before (prestimulus) and after (poststimulus) a mechanical predator disturbance.

**Additional file 2.** All data generated and analyzed in this study.

## Data Availability

The data and materials supporting the conclusions of this article are included within the article and its Additional files 1, 2.
