# Supplementary material for: Paternal care effects outweigh gamete-mediated and personal environment effects during the transgenerational estimation of risk in fathead minnows
Source: BMC Ecol Evol. 2021 Oct 11;21:187. doi: 10.1186/s12862-021-01919-1 (PMC8507329; doi:10.1186/s12862-021-01919-1)
Supplement: Supplementary file 1 — Additional file 1. Section 1. Breeding, rearing and alarm cue exposure protocol. Section 2. Supporting results: Variation in parental care intensity. Section 3. Supporting results: Variation in the change in shoaling density. Section 4. Supporting discussion: Other factors affecting parental care intensity. Table S1. Full and final linear mixed-effect models analysing the factors explaining variation in Pimephales promelas parental care intensity, with risk treatment as the fixed effect of interest. Table S2. Full and final linear mixed-effect models analysing the factors explaining variation in average body size within shoals and within-shoal variation in body size, respectively. Table S3. Impact of average fish size within shoals and shoal heterogeneity on prestimulus shoaling density and the change in shoaling density when density is measured in a absolute distances, i.e., centimeters and b relative distances, i.e., # of body lengths. Fig. S1. Average (over a 10-min period) interindividual distances (mean ± SE) of 39-day old fathead minnows (Pimephales promelas) before (prestimulus) and after (poststimulus) a mechanical predator disturbance. [file 12862_2021_1919_MOESM1_ESM.docx]

**Additional file 1**

**1. Breeding, rearing and alarm cue exposure protocol**

As parental individuals, we used 40 (20 high-risk, 20 low-risk) male and 41 female (20 high-risk, 21 low-risk) *Pimephales promelas* derived from a predator-free laboratory stock population from the Aquatic Toxicology Research Facility, University of Saskatchewan, Saskatoon, Canada. They had previously purchased the population from a commercial supplier (Aquatic Research Organisms Inc., Hampton, USA) that obtained first individuals from the United States Environmental Protection Agency, Ohio in 1985. These fish were then further bred in captivity and in 1989, 1993, 1999, 2005 and 2017, they were outcrossed with fish from other commercial fish hatcheries and research laboratories in Arkansas, Colorado, New York, and Virginia. While one may assume that such laboratory fish populations may have a lower propensity for plasticity, previous studies on minnows from the same population [1-3] as well as on goldfish that have been bred in captivity for over 1000 years [4, 5] suggest that antipredator phenotypic plasticity is retained even after such long periods, likely because predation is a strong selection pressure. Parental fish originated from a split-clutch design where they were kept in family groups and had been from birth onwards, for 5 days per week, exposed to high perceived risk in the form of conspecific alarm cues in a concentration of 3.302 x 10^-6^ cm² skin / l within tanks or to low perceived risk in the form of a water control [see 1 for more details]. All parental individuals here were previously studied in regard to their within-generational antipredator plasticity [1-3], but low-risk and high-risk individuals were never treated differently during these experiments, and fish were never exposed to alarm cues during trials. From April 2018 until September 2018, parental fish were randomly selected as well-developed individuals with breeding coloration from their stock tanks, measured on graph paper to the next millimeter (standard length: from the tip of the snout to the base of the tail fin) and weighed to the next milligram on a digital scale (M-Power AZ153, Sartorius, Göttingen, Germany). Then, we formed outbred pairs and transferred them into 34.5 x 27.0 x 18.5 cm (L x W x H) tanks (PC90 10l with LID90I-4 blue poly lid, Pentair Aquatic Eco-Systems, Atlanta, USA), each containing 375 ml of gravel (mean±SD weight: 540 ± 15 g), an airstone and two breeding tiles (halved PVC pipe pieces, diameter 9.5 cm, length 7.5 cm) each. In these tanks, pairs were kept at seasonally fluctuating temperatures that were kept consistent between risk treatments (median 24.8 °C, IQR 2.1 °C, range 16.5 – 29.8 °C; within-tank variation: IQR 2.2 °C , across-tank variation: IQR 0.475 °C) in a 16:8 h light:dark cycle (from 6 am to 10 pm), fed twice per day with bloodworms *ad libitum* and received a 30% water change every day. We did not continue any of the parental risk treatments within breeding tanks so as to prevent the exposure of embryos within eggs to any residual risk-related cues. Furthermore, we always set up all parental risk treatment combinations concurrently to prevent parental age effects from confounding our results. In total, we set up 43 pairs of 4 different parental combinations (10-12 pairs per parental combination, see Fig. 1 of the main manuscript). Twice per day (at 11 AM and 5 PM), we checked for the presence of eggs. If eggs were present, we removed the breeding tile and photographed it underwater to assess egg numbers. Then, we used a moist rubber glove and gently rubbed eggs off the tile with circle motions; all eggs were removed for the eight risk treatment combinations that did not include parental care. We also had to remove a part of each clutch that received parental care because one of the main aims of the present manuscript is to control for genetic and clutch effects by using a split-clutch approach (i.e., comparing effects of risk-related parental care vs. no parental care/effects of different personal risk environments). This necessitated removing enough eggs to raise a sufficient amount of juveniles from the same clutch in the absence of parental care and allowed only for a standardization of the number of eggs removed relative to original clutch size (small clutches consisting of <40 eggs were not used). For the two treatments where parents took care of their own offspring, we removed approximately 40-60% of all eggs from the tile and returned the remaining eggs to the caring parents (median 53.4 %, IQR 20.9 %, range 29.1 – 72.4 % of original clutch size). A clutch size reduction of 50 % is known to reduce *P. promelas* parental care intensity by approximately 20-30 % [6].Tiles were returned on top of a Ø 10 cm petri dish covered with a 3x3mm plastic mesh in order avoid cannibalization on newly hatched juveniles. Two of the four parental treatments were cross-fostering treatments. Here, immediately after removing approximately half of the eggs, we swapped the tiles between parents from the opposing treatments as long as they had laid eggs within 12 hours of each other, leading to an increase or decrease in clutch size (median 51.5 %, IQR 19.1 %, range 756.2 % – 87.2 % of original clutch size; the limited number of pairs that reproduced on the same day did not allow for standardization of clutch sizes). Neither the number of eggs that parents cared for (Kruskal-Wallis test, **χ**²=6.425, df=3, p=0.093) nor the proportion of eggs that changed as a consequence of removing eggs or switching clutches (Kruskal-Wallis test, **χ**²=2.481, df=3, p=0.479) differed significantly between the four parental care treatments. Removed eggs were split into two equally sized groups, which were randomly assigned to either the high- or low-risk treatment. Groups of eggs were each placed into a Ø 12 cm plastic cup filled with 500 ml water that contained a gently bubbling airstone, 80% water changes were conducted daily. Temperatures were kept constant across cups and risk treatments but fluctuated seasonally within single cups: median 22.9 °C, IQR 1.9 °C, range 18.1 – 26.2 °C). After the eggs hatched, we split fish into two to three replicates of 10 fry each and transferred them into new cups. For the parental care treatments, when the removed eggs of the same clutch within cups started to hatch (at the 4^th^ day after laying), we always removed the tile containing the other part of the clutch from the parental tank, moved it to a plastic cup and let offspring hatch, after which we likewise separated them out in groups of 10 fry each. After hatching, fish from the eight no-care treatments received low-risk and high-risk treatments according to the experimental design; fish from the four parental care treatments consistently received low-risk treatments only (see Fig. 1 of the main manuscript). All additional fry were used for a back-up replicate that was density-unmatched (median 25.5 fry, IQR 37.75 fry, range 0 – 237 fry) and raised in 20 x 30 x 12.5 cm (L x W x H) tanks (5 l water volume) but otherwise treated the same as the first two replicates. In the case of mortality during the first 39 days after hatching, we replaced dead individuals with a random fish from the back-up replicate; fish mortality in this period was similar across risk treatments (median 0 %, IQR 0 – 9.1 %, range 0 – 60 %; Kruskal-Wallis test: **χ**² = 10.122, p = 0.519). Provided food amounts were sequentially increased over development: 1-3d: 1µl sieved *Artemia* nauplii/fish; 4-18d: 10µl sieved *Artemia* nauplii /fish; 18-39d: 20 µl sieved *Artemia* nauplii /fish. Throughout rearing, fish in different tanks did not have visual or olfactory contact. In total, we collected 69 clutches (11-23 clutches per parental combination) from 43 different pairs and used them to generate 281 shoals of 10 fish each (12 risk treatment combinations of 18-43 shoals each; eight of these combinations were without parental care whereas four of them involved parental care; see Fig. 1 of the main manuscript).

**2. Supporting results: Variation in parental care intensity**

In addition to the other factors that shaped parental care intensity (see main manuscript and Table S1), the risk treatment likewise significantly impacted parental care intensity (R²=0.135 [0.034, 0.355], F_3,37.517_=4.433, p=0.009). Males provided the greatest intensity of care in the risk treatments where they took care of their own eggs: biparental low-risk clutches that received low-risk care (87.2±2.8% time spent with the clutch) and in biparental high-risk clutches that received high-risk care (88.2±2.5%). In the other two treatments, care levels were lower (biparental low-risk, high-risk care: 70.8 ± 5.1%; biparental high-risk, low-risk care: 80.4±3.0%). The different risk treatments within own or adopted clutches did not differ significantly from each other (all p≥0.763, d_Cohen_=0.059 [-0.376, 0.493] to 0.368 [-0.097, 0.833]). However, when comparing risk treatments across own or adopted clutches, both tendential and significant effects emerged (all p≤0.055, d_Cohen_=0.369 [-0.082, 0.820] to 0.702 [-0.240, 1.163]). These results suggest that the observable effect of risk treatment is a by-product of own clutches receiving more care than adopted ones. Consequently, using ‘care type’ (own/adopted) as a fixed effect rather than ‘risk treatment’ was found to generate a better model fit (see Table 1 of the main manuscript).

**Table S1**: Full and final linear mixed-effect models analysing the factors explaining variation in *Pimephales promelas* parental care intensity, with risk treatment as the fixed effect of interest. Variation in parental care (i.e., proportion of time spent next to the clutch) was Yeo-Johnson transformed before analysis.

|  | df_Numerator_ for fixed effects | df_Denominator_ for fixed effects | F for fixed effects,  χ² for random effects | *P* |
| --- | --- | --- | --- | --- |
| **Variation in parental care** |  |  |  |  |
| *Full model* |  |  |  |  |
| risk treatment × day of care | 3 | 54.299 | 1.617 | 0.196 |
| risk treatment × clutch size | 3 | 35.949 | 1.208 | 0.321 |
| risk treatment × proportional change in clutch size | 3 | 36.916 | 1.599 | 0.206 |
| day of care | 1 | 20.391 | 6.588 | 0.018 |
| clutch size | 1 | 35.934 | 3.418 | 0.073 |
| proportional change in clutch size | 1 | 40.305 | 3.899 | 0.055 |
| risk treatment | 3 | 35.644 | 1.739 | 0.177 |
| caring parent ID × family |  |  | 2.960 | 0.228 |
| family |  |  | 1.197 | 0.550 |
|  |  |  |  |  |
| *Final model* |  |  |  |  |
| day of care | 1 | 31.004 | 5.914 | 0.021 |
| proportional change in clutch size | 1 | 39.925 | 5.946 | 0.019 |
| risk treatment | 3 | 37.514 | 4.433 | 0.009 |
| caring parent ID × family |  |  | 4.896 | 0.086 |
| family |  |  | 1.616 | 0.446 |

**3. Supporting results:** **Variation in the change in shoaling density**

During model reduction of the full model that analyzed variation in the change in shoaling density, we found that in addition to the described effects, the relationship between shoal average body size and the change in shoaling density differed between treatments (risk treatment × average size within shoals, R²=0.069 [0.056, 0.222], F_11,281_=2.160, p=0.017). However, investigating every risk treatment separately (with shoal homogeneity as a covariate), average size within shoals never impacted significantly on the change in shoaling density in any risk treatment (all p > 0.090). Hence, this interaction is unlikely to confound our results. As all other interactions turned out to be nonsignificant during model reduction, we re-ran the model reduction based on a full model that did not contain any interaction terms so as to generate the final model shown in Table 2 of the main manuscript.

**4. Supporting discussion: Other factors affecting parental care intensity**

First, parental care intensity was on average 12.4% lower for adopted clutches, which is in accordance with a previous study on *P. promelas* that found fathers to provide 19-25 % less care for adopted clutches [7]. Although this result suggests that *P. promelas* are capable of embryronic kin recognition as other fish species [8], the exact mechanism of how they discriminate between own and foreign eggs remains unknown. Although adopting clutches facilitates the attraction of mates [9], fitness-related costs associated with taking care of foreign embryos is likely to underlie the lower care levels. Second, parental care intensity correlates positively with the proportional change in clutch size due to experimental manipulation, which explains an ~15 % decrease in parental care intensity on average over the full range of 756.2 % – 72.4 % changes to original clutch size. This observed effect is much weaker than in previous studies in *P. promelas* where clutch size reductions of 50% caused a 20-30 % decrease in parental care intensity [6]. Nevertheless, this result is in accordance with previously described links between parental care intensity and clutch size manipulation across taxa [10-12]. Lastly, paternal care intensity correlated positively with clutch age, with an on average ~6.25% increase in care over the 4-day care period. This is likely related to the higher fitness cost from losing a clutch that received previous time and energy investment. Lower levels of parental care have been suggested to be linked to lower egg survival in *P. promelas*, but a direct causal link remains to be established [6, 7].

**Table S2**: Full and final linear mixed-effect models analysing the factors explaining variation in average body size within shoals and within-shoal variation in body size, respectively

|  | df_Numerator_ for fixed effects | df_Denominator_ for fixed effects | F for fixed effects,  χ² for random effects | *P* |
| --- | --- | --- | --- | --- |
| **Average body size within shoals** |  |  |  |  |
| *Full model* |  |  |  |  |
| risk treatment × paternal condition | 11 | 126.374 | 0.460 | 0.925 |
| risk treatment × maternal condition | 11 | 120.502 | 1.045 | 0.412 |
| risk treatment × original clutch size | 11 | 214.965 | 0.973 | 0.472 |
| paternal condition | 1 | 46.271 | 0.509 | 0.479 |
| maternal condition | 1 | 43.231 | 0.106 | 0.747 |
| original clutch size | 1 | 152.246 | 0.713 | 0.400 |
| risk treatment | 11 | 125.245 | 0.957 | 0.489 |
| family |  |  | 113.750 | <0.001 |
|  |  |  |  |  |
| *Final model* |  |  |  |  |
| family |  |  | 116.46 | <0.001 |
|  |  |  |  |  |
| **Within-shoal variation in body size (i.e., shoal homogeneity)** |  |  |  |  |
|  |  |  |  |  |
| *Full model* |  |  |  |  |
| risk treatment × paternal condition | 11 | 138.532 | 1.219 | 0.280 |
| risk treatment × maternal condition | 11 | 131.620 | 0.713 | 0.725 |
| risk treatment × original clutch size | 11 | 222.938 | 0.670 | 0.766 |
| paternal condition | 1 | 48.875 | 0.786 | 0.380 |
| maternal condition | 1 | 45.133 | 3.254 | 0.078 |
| original clutch size | 1 | 160.794 | 1.163 | 0.283 |
| risk treatment | 11 | 143.861 | 0.772 | 0.668 |
| family |  |  | 75.205 | <0.001 |
|  |  |  |  |  |
| *Final model* |  |  |  |  |
| family |  |  | 101.03 | <0.001 |

**Table S3**: Impact of average fish size within shoals and shoal heterogeneity on prestimulus shoaling density and the change in shoaling density when density is measured in a) absolute distances, *i.e.,* centimeters and b) relative distances, *i.e.,* # of body lengths. Partial R²-values with 95% confidence intervals (CI), F-values and p-values are derived from linear mixed-effect models containing only average fish size within shoals and shoal heterogeneity as fixed effects, and family identity as a random intercept.

|  | **F** | **R² [95 % CI]** | **p** |
| --- | --- | --- | --- |
| **Prestimulus shoaling density** |  |  |  |
| *average fish size within shoals* |  |  |  |
| absolute distances (cm) | 5.837 | 0.019 [0, 0.081] | 0.016 |
| relative distances (“# body lengths”) | 14.543 | 0.061 [0,0.157] | <0.001 |
| *shoal heterogeneity* |  |  |  |
| absolute distances (cm) | 7.731 | 0.026 [0,0.087] | 0.006 |
| relative distances (“# body lengths”) | 4.924 | 0.023 [0,0.124] | 0.027 |
|  |  |  |  |
| **Change in shoaling density** |  |  |  |
| *average fish size within shoals* |  |  |  |
| absolute distances (cm) | 0.333 | 0.001 [0,0.048] | 0.565 |
| relative distances (“# body lengths”) | 0.108 | 0.001 [0,0.041] | 0.743 |
| *shoal heterogeneity* |  |  |  |
| absolute distances (cm) | 4.758 | 0.018 [0.001,0.065] | 0.031 |
| relative distances (“# body lengths”) | 1.554 | 0.004 [0, 0.045] | 0.214 |

**
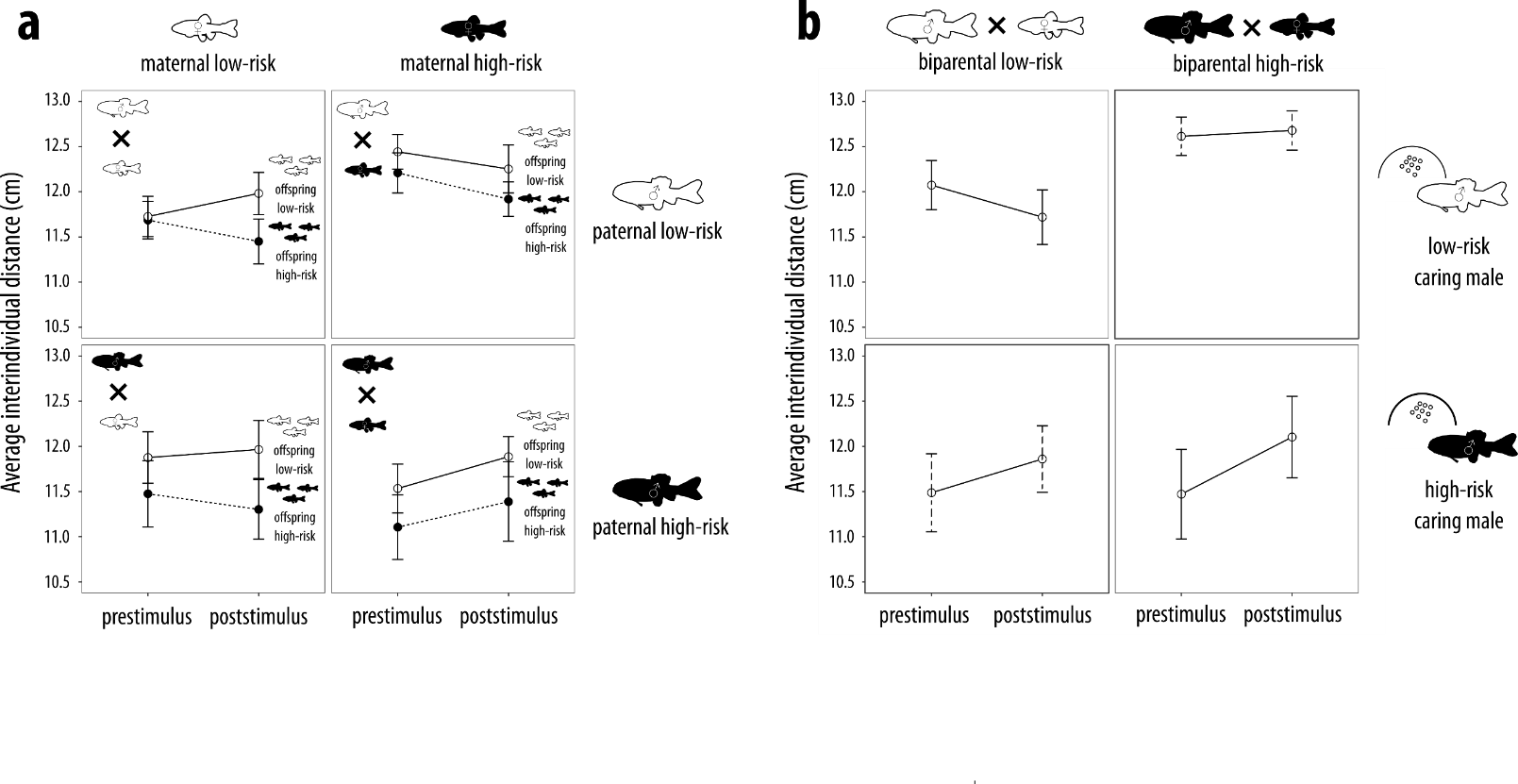
**

**Fig. S1**: Average (over a 10-min period) interindividual distances (mean ± SE) of 39-day old fathead minnows (*Pimephales promelas*) before (prestimulus) and after (poststimulus) a mechanical predator disturbance. Showcased are a) maternal × paternal × offspring risk interactions and b) biparental × caring parent risk interactions. In a), empty dots represent offspring low-risk whereas filled dots represent offspring high-risk environments. In b), offspring were always exposed to low-risk environments only, hence no filled dots are present. Within fish drawings, black fish indicate individuals that were from birth onwards exposed to conspecific alarm cues, white fish refer to the ones that instead received a water control treatment; for more detail see Fig. 1 of the main manuscript. Dashed error bars in b) highlight the treatments that were cross-fostered.

**References**

1. Meuthen D, Ferrari MCO, Lane T, Chivers DP: High background risk induces risk allocation rather than generalized neophobia in the fathead minnow. Behav Ecol. 2019; 30:1416-1424.

2. Meuthen D, Ferrari MCO, Lane T, Chivers DP: Predation risk induces age- and sex-specific morphological plastic responses in the fathead minnow *Pimephales promelas*. Sci Rep. 2019; 9:15378.

3. Meuthen D, Ferrari MCO, Lane T, Chivers DP: Plasticity of boldness: high perceived risk eliminates a relationship between boldness and body size in fathead minnows. Anim Behav. 2019; 147:25-32.

4. Chivers DP, Zhao X, Ferrari MCO: Linking morphological and behavioural defences: Prey fish detect the morphology of conspecifics in the odour signature of their predators. Ethology. 2007; 113:733-739.

5. Chivers DP, Zhao XX, Brown GE, Marchant TA, Ferrari MCO: Predator-induced changes in morphology of a prey fish: the effects of food level and temporal frequency of predation risk. Evol Ecol. 2008; 22:561-574.

6. Sargent RC: Paternal care and egg survival both increase with clutch size in the fathead minnow, *Pimephales promelas*. Behav Ecol Sociobiol. 1988; 23:33-37.

7. Sargent RC: Allopaternal care in the fathead minnow, *Pimephales promelas*: stepfathers discriminate against their adopted eggs. Behav Ecol Sociobiol. 1989; 25:379-385.

8. Wells MW, Wright PA: Do not eat your kids: embryonic kin recognition in an amphibious fish. Behav Ecol Sociobiol. 2017; 71:140.

9. Unger LM, Sargent RC: Allopaternal care in the fathead minnow, *Pimephales promelas*: females prefer males with eggs. Behav Ecol Sociobiol. 1988; 23:27-32.

10. Sanz JJ: Clutch size manipulation in the pied flycatcher: effects on nestling growth, parental care and moult. J Avian Biol. 1997; 28:157-162.

11. Verhulst S, Tinbergen JM: Clutch size and parental effort in the Great Tit *Parus major*. Ardea. 1997; 85:111-126.

12. Lavery RJ, Keenleyside MHA: Parental investment of a biparental cichlid fish, *Cichlasoma nigrofasciatum*, in relation to brood size and past investment. Anim Behav. 1990; 40:1128-1137.
